# An unsupervised learning approach to identify novel signatures of health and disease from multimodal data

**DOI:** 10.1186/s13073-019-0705-z

**Published:** 2020-01-10

**Authors:** Ilan Shomorony, Elizabeth T. Cirulli, Lei Huang, Lori A. Napier, Robyn R. Heister, Michael Hicks, Isaac V. Cohen, Hung-Chun Yu, Christine Leon Swisher, Natalie M. Schenker-Ahmed, Weizhong Li, Karen E. Nelson, Pamila Brar, Andrew M. Kahn, Timothy D. Spector, C. Thomas Caskey, J. Craig Venter, David S. Karow, Ewen F. Kirkness, Naisha Shah

**Affiliations:** 1 Human Longevity, Inc., San Diego, CA 92121 USA; 2 Electrical and Computer Engineering, University of Illinois at Urbana-Champaign, Urbana, IL 61820 USA; 3 J. Craig Venter Institute, La Jolla, CA 92037 USA; 4 Division of Cardiovascular Medicine, School of Medicine, University of California San Diego, La Jolla, CA 92093 USA; 5 Department of Twin Research and Genetic Epidemiology, King’s College London, London, UK; 6 Molecular and Human Genetics, Baylor College of Medicine, Houston, TX 77030 USA

**Keywords:** Multimodal, Preventative medicine, Metabolomics, Cardiometabolic syndrome, Unsupervised machine learning, Network analysis

## Abstract

**Background:**

Modern medicine is rapidly moving towards a data-driven paradigm based on comprehensive multimodal health assessments. Integrated analysis of data from different modalities has the potential of uncovering novel biomarkers and disease signatures.

**Methods:**

We collected 1385 data features from diverse modalities, including metabolome, microbiome, genetics, and advanced imaging, from 1253 individuals and from a longitudinal validation cohort of 1083 individuals. We utilized a combination of unsupervised machine learning methods to identify multimodal biomarker signatures of health and disease risk.

**Results:**

Our method identified a set of cardiometabolic biomarkers that goes beyond standard clinical biomarkers. Stratification of individuals based on the signatures of these biomarkers identified distinct subsets of individuals with similar health statuses. Subset membership was a better predictor for diabetes than established clinical biomarkers such as glucose, insulin resistance, and body mass index. The novel biomarkers in the diabetes signature included 1-stearoyl-2-dihomo-linolenoyl-GPC and 1-(1-enyl-palmitoyl)-2-oleoyl-GPC. Another metabolite, cinnamoylglycine, was identified as a potential biomarker for both gut microbiome health and lean mass percentage. We identified potential early signatures for hypertension and a poor metabolic health outcome. Additionally, we found novel associations between a uremic toxin, *p-*cresol sulfate, and the abundance of the microbiome genera *Intestinimonas* and an unclassified genus in the *Erysipelotrichaceae* family.

**Conclusions:**

Our methodology and results demonstrate the potential of multimodal data integration, from the identification of novel biomarker signatures to a data-driven stratification of individuals into disease subtypes and stages—an essential step towards personalized, preventative health risk assessment.

## Background

Despite the enormous US healthcare spending of $3.3 trillion in 2016 [1], one in three individuals aged 50–74 years die prematurely from major age-related chronic diseases [2–4]. Challenging the status quo of our reactive healthcare, preventative medicine offers an alternative means to better health for lower cost [5]. One approach to move beyond traditional medicine to more predictive, preventive practices is via systems medicine. As defined by Hood and Flores [6], systems medicine is the application of systems biology to the challenges of human health and disease. An interdisciplinary approach that measures, integrates, analyzes, and interprets a variety of clinical and non-clinical data is critical for a deeper understanding of the mechanisms that determine health and disease states. Significant computation and statistical analysis are essential to sift through large, diverse datasets and search for patterns, whether related to specified biological processes or to stratify complex diseases into distinct subsets for health assessment.

Recent studies have shown the utility of collecting and analyzing diverse high-throughput data using unsupervised computational methods for more comprehensive insights into biological systems. Argelaguet et al. [7] showed a need for such integrated analysis by introducing a computational framework of unsupervised integration of heterogeneous data and showed its utility by identifying major drivers of variation in chronic lymphocytic leukemia. Price et al. [8] revealed communities of related analytes associated with diseases using unsupervised network analysis on a multimodal dataset.

In our previous work [4], we introduced a platform of deep quantitative multimodal phenotyping that seeks to provide a comprehensive, predictive, preventative, and personalized assessment of an individual’s health status. The offered multimodal assays include whole genome sequencing, advanced imaging, metagenomic sequencing, metabolome, and clinical labs. In addition, medical history and family history were also collected from the individuals. The collected data is used to screen individuals for precision medicine. This includes identification of clinically significant pathogenic variants and clinical summaries from advanced imaging and other clinical testing [4]. This platform provides critical data not only to identify previously undiagnosed disease states but also to identify early disease biomarkers. Here, we present an analysis of the multimodal datasets that were collected for 1253 self-assessed healthy adults and an independent validation dataset consisting of 1083 adults with longitudinal data. To the best of our knowledge, this is the largest cohort with such a wide range of data modalities analyzed to date.

In this study, expanding on the unsupervised approaches described above, we perform a comprehensive analysis with the aims to not only find novel patterns in disease risk but also to stratify individuals into health states using newly identified biomarker signatures. We performed a combination of machine learning analyses, including cross-modality associations, network analysis to identify modules and their key biomarkers, and stratification of individuals into distinct health risk groups and their longitudinal outcomes. By doing so, we showcase a framework to assess an individual’s disease risk by identifying signatures of health and disease through unsupervised learning on multimodal data.

## Methods

### Data collection and data features

For the study, we collected data from 1253 self-assessed healthy individuals in our clinical research facility. We used several tools and techniques referred to as *modalities* to collect the data. The modalities included whole genome sequencing (WGS), microbiome sequencing, global metabolome, insulin resistance (IR as defined by Cobb et al. [9]) and impaired glucose intolerance (IGT as defined by Cobb et al. [10]), laboratory developed tests (Quantose™), whole body and brain magnetic resonance imaging (MRI), dual-energy x-ray absorptiometry (DEXA), computed tomography (CT) scan, routine clinical laboratory tests, personal/family history of disease and medication, and vitals/anthropometric measurements. Data collection has been described in detail in our previous manuscript on the first 209 individuals enrolled in a precision medicine study [4]. In addition to the modalities described in the previous study, we have now included CT scan and microbiome sequencing [11]. Not all data was collected on all individuals. The number of individuals and the number of features per modality are summarized in Additional file 1: Table S1.

We performed CT scans on individuals over the age of 35 years. Patients were scanned during a single breath-hold using a 64-slice GE Healthcare EVO Revolution scanner (GE Healthcare, Milwaukee, Wisconsin). Gated axial scans with 2.5 mm slice thickness were performed using a tube energy of 120 kVp and the tube current adjusted for individuals’ body mass index. Images were subsequently analyzed using an AW VolumeShare 7 workstation (GE Healthcare, Milwaukee, Wisconsin), and regions of coronary calcification were manually identified in order to compute Coronary Artery Calcium (CAC) Agatston scores [12]. We used Multi-Ethnic Study of Atherosclerosis (MESA) [13] reference CAC values to calculate the percentile of calcification for each individual matched for age, sex, and ethnicity.

For microbiome sequencing, we performed whole genome sequencing on stool samples to analyze the microbial communities [11]. For this modality, the features included species richness, species diversity, the fraction of human DNA, *Proteobacteria*, and the abundance of 72 genera [11, 14]. Microbiome species richness was defined as the number of species present at a relative abundance greater than 10^−4^. Microbiome species diversity was defined as the Shannon entropy of the taxon abundance vector [15].

Whole genome sequencing data was used to compute the following features: polygenic risk scores (PRS) [16] for 51 diseases and traits, HLA type [17], 30 known short tandem repeats (STR) disease loci [18], and known rare pathogenic variants from ClinVar (set 1 and set 2 from Shah et al. [19]). We also computed ancestry using the method described by Telenti et al. [20] from WGS data.

### Data pre-processing

To satisfy the normality assumption of the statistical tests used in the analysis, we first performed data transformation on certain features (described below) and then adjusted for covariates. This order of data pre-processing has been shown to avoid introducing bias [21]. To address the non-Gaussian distributions of various features from several modalities, we utilized a rank-based inverse normal transformation [22]. We applied this transformation to all microbiome abundance data, as these features exhibit non-Gaussian distributions. The transformation was also applied to other features where more than 40% of the samples had the same value. Several features were correlated with age, sex, and/or ancestry. To remove this correlation, we used multiple linear regression to identify the significantly associated (*p* < 0.01) covariates among age, sex, and the first four principal components representing the ancestry. The feature values were corrected by regressing out the significantly associated covariates.

### Network analysis

We used a combination of methods to build a cross-modality association network, identify densely connected modules within the association network, and then extract the key biomarkers representing each module. More precisely, we first used Spearman’s correlation to identify statistically correlated pairs of cross-modality features. Second, we used the Louvain community detection method to identify distinct modules reflective of biologically functional subnetworks. Third, to identify the key features within a densely connected module, we constructed a sparse network (also known as *Markov network*) using the Graphical Lasso method. Below, we describe these steps in more detail.

We point out that technically one could apply the Graphical Lasso method to the entire dataset in order to produce a Markov network with all the features. However, since within-modality associations tend to be stronger than cross-modality associations, features from each modality have a tendency to cluster together. A resulting network using all features is shown in Additional file 1: Figure S9. Additionally, the Graphical Lasso method requires a complete data matrix, which will lead to imputing values for the missing data for all features (vs. a smaller subset of features in our multistep approach). This can make the results less reliable. By first constructing an association network based only on cross-modality associations (following the approach from Price et al. [8]) and running a community detection algorithm, the resulting communities tend to be multimodal. Thus, we opted to use a combination of network methods to obtain more informative modules with multimodal features.

#### Constructing multimodal correlation modules

We performed Spearman’s correlation analysis and calculated *p* values for each cross-modality pair of features. The correlation was computed on individuals for which both features were present. The correlation was calculated only if at least 30 individuals had data for the pair of features. We selected statistically significant association using the Benjamini-Hochberg [23] approach to limit the false discovery rate to 5%.

The significant associations were used to construct a network where each feature is a *node*, and the association between two feature nodes is an *edge*. The weight of an edge is defined as −log(*p*), where *p* is the *p* value of the corresponding Spearman correlation. Metabolome and clinical lab measured several of the same or similar metabolites. To avoid having the structure of the network mainly driven by strong associations between the metabolome and clinical lab features, we ignored those edges for the identification of the initial modules. To identify densely connected sets of nodes, i.e., “modules” in the network, we used the Louvain algorithm for community detection [24]. The Louvain method is a widely used tool for uncovering community structure from large networks. It seeks to maximize the network *modularity* in a greedy fashion. Initially, each node is seen as its own community. Nodes are then iteratively merged, such that it maximizes the gain in modularity until the modularity can no longer be increased. The resulting “super-nodes” are the communities. For a true representation of molecular features involved in multiple biological functions, we allowed for nodes to belong to multiple modules. More precisely, when a node assigned to a module had at least 20 more significant associations with nodes from another module than it had with its assigned module, then the node was placed in both the modules.

The robustness of the resulting modules was assessed in the following ways (additional details in Additional file 3: Supplementary Notes). The Louvain community detection algorithm was run 300 times with different seeds, which is used to order the nodes for community expansion. We examined the modularity score for each of the runs. Next, we built a consensus matrix [25] by calculating the number of times the same pair of nodes are grouped together in a module, across the 300 runs. Additionally, we calculated consistency score for both sets of key biomarkers identified in the two modules, by counting the average number of times a pair of features were grouped in the same module.

#### Key biomarker selection and Markov network construction

We performed a deeper analysis of the two largest modules identified using the community detection method. Firstly, a list of module-representative features was identified. Specifically, for each module, we ranked the nodes by their eigenvector centrality score to identify the topmost central features. Secondly, a conditional independence network for each of the selected modules was derived. Specifically, we used the central features to construct a sparse network using the Graphical Lasso method [26]. This method estimates the inverse covariance matrix of the selected features using a lasso penalty to induce sparsity. The method does not allow for missing values in the data matrix and assumes a normally distributed data. Thus, the central features used in this method were mean-imputed (only 10% of the matrix required imputation) and converted to Gaussian distributions using the rank-based inverse normal transformation as described in the “Data pre-processing” section. In the resulting conditional independence network (also known as a “Markov network”), the absence of an edge between two features implies that they are conditionally independent given the remaining features in the network. Additional information on Markov network is provided in Additional file 3: Supplemental Notes.

In the Markov network, features that had a connection with at least one cross-modality feature were selected as *key biomarkers* for the downstream analysis. This procedure of selecting key biomarkers ensures that the inherently stronger associations within each modality do not overpower associations that are cross modal, thus avoiding biased representation. Unlike the cross-modality correlation network, in the Markov network, the edges between features from the same modality were included. This allowed for identification of key biomarkers of the underlying biological mechanism regardless of the modality origin.

### Stratifying individuals with similar biomarker signatures

For each selected module, we used the identified key biomarkers to stratify the individuals. Each feature was scaled to have zero mean and unit variance. The missing values were imputed using softImpute [27]. Then, we performed hierarchical clustering on the individuals based on complete linkage and a correlation distance metric. We selected the lowest cutting point of the hierarchical cluster tree such that the resulting clusters would have at least 50 individuals. To access the clustering, we computed for each individual the median distance to each of the seven subsets and identified the closest subset to each individual.

### Statistical associations between clusters and other traits

We compared the rates of disease diagnosis and medication use across the seven cardiometabolic and the seven microbiome richness subsets. Fisher’s exact test was used (using a Monte Carlo simulated *p* value with 1E6 replicates) to test for statistical significance after the Bonferroni correction for multiple tests.

We also compared the individuals in each subset to all individuals not in that subset for each of the 1354 features using a logistic regression. There was thus a separate analysis performed for subset 1 vs. everyone else, subset 2 vs. everyone else, etc. Significant associations were those that survived the Bonferroni correction for multiple tests.

### Validation cohort

For validation of our findings, we utilized 1083 individuals from a study cohort (referred to here as “TwinsUK”) of largely European ancestry female twins enrolled in the TwinsUK registry, a British national register of adult twins [28]. The cohort included data from WGS, metabolome, microbiome, DEXA, clinical blood laboratory tests, and personal history of disease and medication. The data from the modalities was collected from three longitudinal visits over the course of a median of 13 years. To capture a population with adequate sample sizes for the overlapping modalities used in the present study, we restricted our analysis to data from visit 2 (referred here as “baseline”) and visit 3 (referred here as “follow-up”). Microbiome samples were only collected at visit 3. The median age at visit 2 was 51, range 41–79. To be included in the analysis, phenotyping measurements were required to be collected within 90 days of the metabolome draw for each visit, or within 6 months for microbiome. For the validation of metabolome and microbiome correlations, we used only one of the twins to avoid bias from relatedness, totaling 538 individuals. For the cardiometabolic module analysis, we imputed liver fat, gamma-glutamyl transferase (GGT), IGT, IR, and glucose using regularized linear regression with L1 penalty.

### Data pre-processing and assignment for validation cohort

The data was pre-processed using the same correction for age, sex, and ancestry that was used for the main cohort. Specifically, we used the regression coefficients learned during the pre-processing of the study cohort dataset to correct the data in the validation cohort. The corrected data from the validation cohort was then mapped to the individual subsets as follows. For each subset, the median for each of the key biomarkers within the subset was computed, giving rise to a “representative signature” for that subset. For each individual in the validation cohort, correlation distances to each of the representative signatures were computed, and the individual was assigned to the closest one. Note that correlation distances were used since the hierarchical clustering of the study cohort was conducted using correlation distance.

## Results

**Fig. 1:**
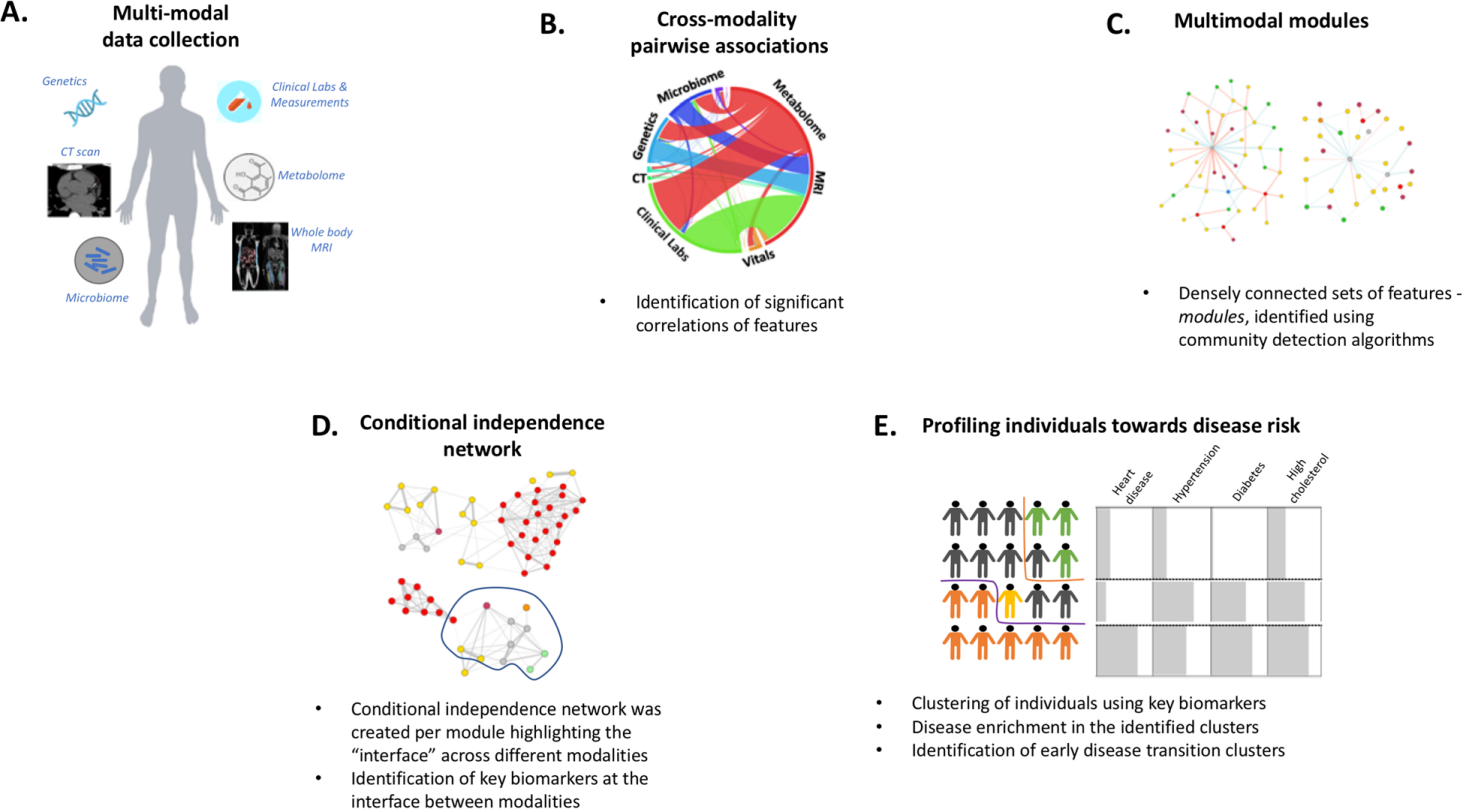
**a** In the study, we collected multimodal data (*n* = 1385 features) from 1253 individuals. **b** We analyzed the data by performing cross-modality associations between features after correcting for age, sex, and ancestry. **c** Using the associations, we performed community detection analysis and found modules of densely connected features. **d** To reduce the number of indirect associations and identify key biomarker features, we performed conditional independence network analysis (also referred to as a *Markov network*). **e** Using the identified key biomarkers, we clustered individuals into distinct groups with similar signatures that are consistent with different health statuses. We characterize the clusters and perform disease risk enrichment analysis

We carried out multimodal tests on 1253 self-assessed healthy adults (median age 53; 63% male; 71% European ancestry) using our genomic and deep phenotyping platform [4]. The modalities included whole genome sequencing, metabolome, microbiome, advanced imaging, and clinical tests. We derived 1385 features from the collected data (Additional file 1: Table S1; Additional file 3: Supplementary Notes). To extract patterns indicative of biological mechanisms, we applied machine learning methods to this heterogeneous dataset. Specifically, we performed (1) correlation analysis to identify significant associations between cross-modal features, (2) network analysis to identify modules and their biomarker signatures representative of the underlying biological systems, and (3) cluster analysis to stratify individuals into distinct subsets using the identified signatures that are consistent with different health status (Fig. 1; Additional file 3: Supplemental Notes). We further characterized the subsets and examined disease risk using individuals’ personal history. We used an independent cohort of 1083 females (TwinsUK) to validate our findings and to assess associations with longitudinal disease diagnosis data.

### Multimodal correlations and network analysis

**Fig. 2:**
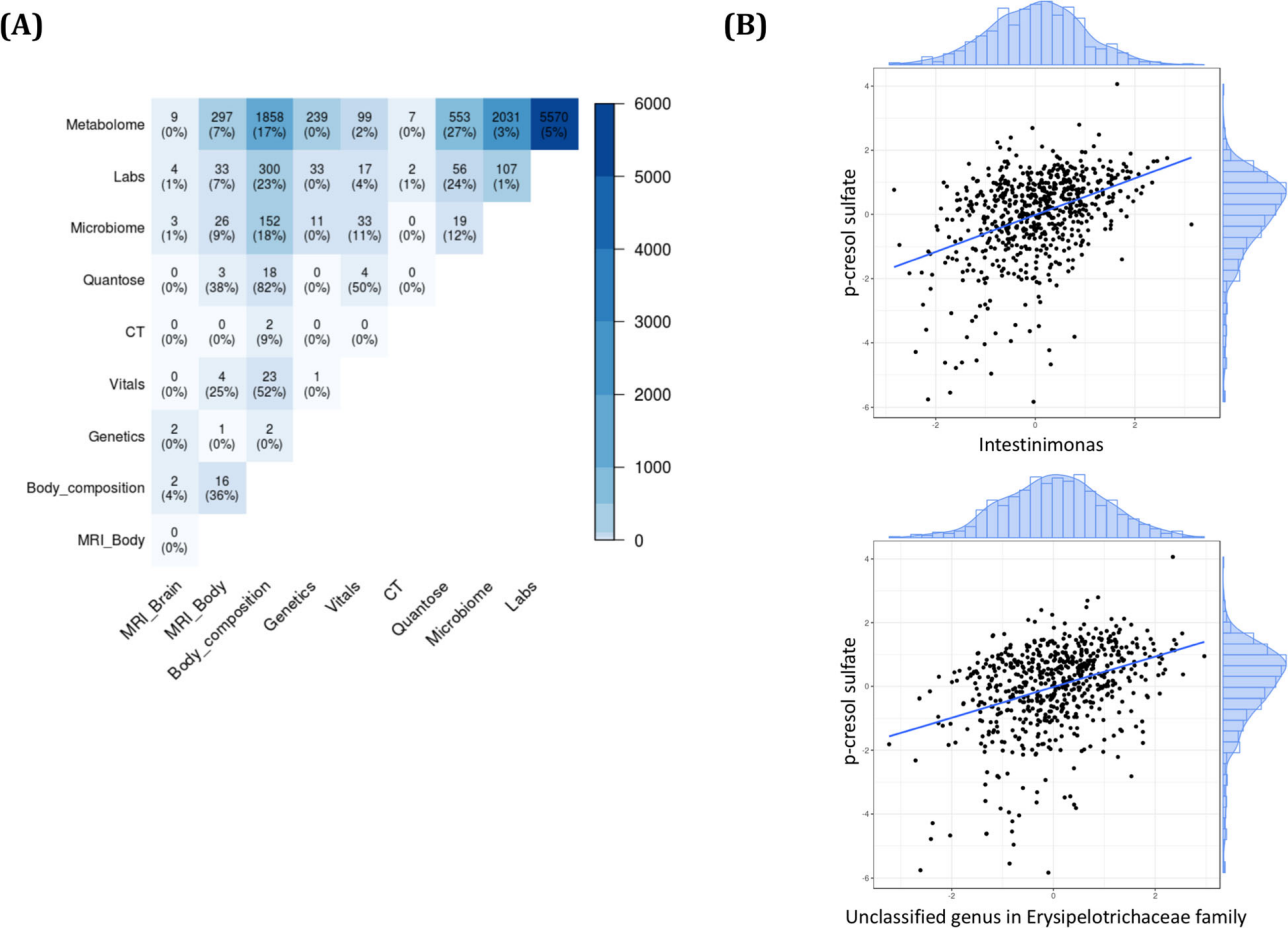
The number of significant cross-modality correlations for each pair of modalities is shown (**a**). The percentages shown are the proportion of correlations that were significant out of all possible pairwise associations between the modality pair. **b** Associations between *p*-cresol sulfate metabolite and (top) abundance of *Intestinimonas* genus, and (bottom) an abundance of unclassified genus in *Erysipelotrichaceae* family

We calculated correlations for each cross-modality pair of normalized features and selected a list of 11,537 statistically significant associations out of 427,415 total cross-modality comparisons (FDR < 0.05; see the “Methods” section). The largest number of significant associations (*n* = 5570) was observed between metabolome and clinical lab features. Of all the possible correlations between features from the two modalities, these significant associations accounted for 5% (Fig. 2a). The second largest number of significant associations was between the metabolome and microbiome features (*n* = 2031; 3%), followed by metabolome and body composition features (*n* = 1858; 17%). We discuss some of these associations below. Additionally, some of the important findings from metabolome and body composition have been discussed in Cirulli et al.

The most significant associations, apart from those between metabolome and lab features, were expected correlations supporting well-established prior clinical research (see Additional file 3: Supplementary Notes). Additionally, we observed novel associations between the metabolite *p*-cresol sulfate (pCS) and the microbiome genus *Intestinimonas* as well as an unclassified genus in the *Erysipelotrichaceae* family (*p* = 2.92E−24 and *p* = 2.98E−20, respectively; Fig. 2b). Other known microbiome features associated with pCS were also observed [29–31]. This included associations with species diversity (*p* = 6.54E−19) and several genera (*Pseudoflavonifractor*, *Anaerotruncus*, *Subdoligranulum*, and *Ruminiclostridium*) in the *Ruminococcaceae* family (*p* = 9.52E−32, *p* = 1.39E−23, *p* = 9.48E−19, and *p* = 3.26E−11, respectively). These associations were validated in the independent TwinsUK cohort (see the “Methods” section; Additional file 1: Table S2).

The significant associations were used to construct a network with features as nodes and feature associations as edges. Using a community detection method, sets of highly connected features (referred to as *modules*) were then identified (see the “Methods” section). Intuitively, the modules should group together features that are biologically related, indicative of biologically functional subnetworks. The result was numerous small modules and two modules that had far larger numbers of features (*n* > 100 each). The largest was a *cardiometabolic module* containing many markers associated with cardiac disease and metabolic syndrome, similar to a module previously observed by Price et al. [8]. The second largest module was predominantly composed of microbiome taxa abundance and several metabolites that are known to be biomarkers for gut microbiome diversity. We refer to this module as the *microbiome richness module*. The modules were tested for their robustness. The average modularity score was 0.37 (Additional file 1: Figure S1), and the consistency score was > 0.80 (Additional file 3: Supplemental Notes; Additional file 1: Figure S2). Next, we present further detailed analysis on these two largest modules.

### Cardiometabolic module

The largest module in the association network contained 355 nodes from clinical labs, metabolome, quantose, CT, microbiome, vitals, genetics, MRI-body, and body composition data modalities. The most central features in the module were identified using an eigenvector centrality score (see the “Methods” section). These features included visceral adipose tissue mass, BMI, liver fat percentage, lean mass percentile, glucose levels, blood pressure (BP), triglycerides levels, IR score, several lipid metabolites, and several microbiome genera, including butyrate-producing bacterial genera such as *Pseudoflavonifractor*, *Butyrivibrio*, *Intestinimonas*, and *Faecalibacterium*. Some of these features are known to be associated with obesity, heart disease, and metabolic syndrome.

#### Network analysis for key biomarker selection

**Fig. 3:**
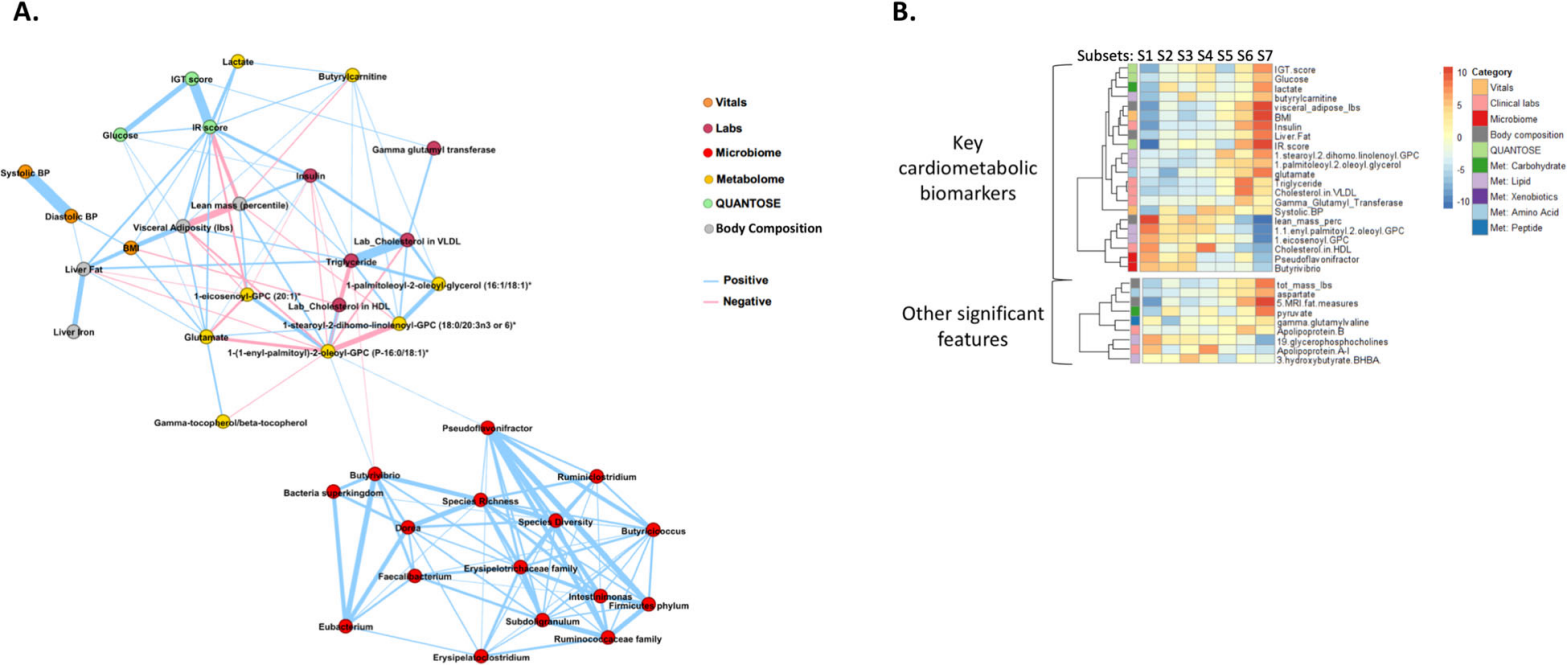
The cardiometabolic module. **a** We built a Markov network to identify the key biomarker features that represent the cardiometabolic module. This network highlights the most important associations after removing edges corresponding to indirect associations. We observed that the microbiome genera *Butyrivibrio* and *Pseudoflavonifractor* are the most relevant microbiome genera in the context of this module that interface with features from other modalities. **b** We clustered individuals using the key biomarkers. The heatmap shows *z*-statistics from logistic regression for an association between each cluster and each feature. The plot on the left shows the 22 key cardiometabolic biomarkers. The plot on the right shows associations that emerged from an analysis against the full set of 1385 features with *p* < 1 × 10^−10^ as well as 3-hydroxybutyrate (BHBA) and Apolipoprotein B because of their particular enrichment in clusters 3 and 6, respectively. Some correlated features have been collapsed, with the mean *z*-statistics displayed; the full set of features can be found in Additional file 1: Figure S1. All of these significant associations showed consistent directions of effect in the TwinsUK cohort (Additional file 2: Table S3); however, the microbiome features and 5 of the glycerophosphocholines were not measured in the TwinsUK cohort and thus could not be assessed for replication. Met, metabolome

To remove redundancy and transitive associations from the module, we created a *Markov network* containing only the associations that were significant after conditioning on all other features (see Additional file 3: Supplemental Notes and the “Methods” section). This process thus captured a more meaningful network of interactions between the features of the module. The resulting cardiometabolic network is shown in Fig. 3a. In the Markov network, features that had a connection with at least one feature from a different modality were selected as *key biomarkers* for downstream analysis. This procedure of selecting key biomarkers ensures that the inherently stronger associations within each modality do not overpower associations that are cross modal, thus avoiding biased representation. For example, the microbiome genera *Butyrivibrio* and *Pseudoflavonifractor* are the only microbiome features that are connected to features from other modalities (the lipid metabolite 1-(1-enyl-palmitoyl)-2-oleoyl-GPC (P-16:0/18:1) and serum triglyceride) and are thus selected as key biomarkers (Fig. 3a). On the other hand, liver iron and gamma-tocopherol/beta-tocopherol are only associated features from their respective modalities and are hence not selected as key biomarkers. A total of 22 key cardiometabolic biomarkers were identified. An exception to this rule was that we replaced diastolic BP with systolic BP. These two features were heavily correlated and essentially interchangeable, but based on an expert opinion, we chose the systolic BP as a better marker for cardiometabolic conditions.

These key biomarkers included established features for cardiac and metabolic conditions (e.g., BMI, BP, glucose levels, and HDL) and also novel biomarkers from metabolome and microbiome (Fig. 3a). High abundance of the microbiome genera *Butyrivibrio* and *Pseudoflavonifractor* was well correlated with features that are generally considered to be correlated with “good” cardiometabolic health (defined using traditional markers such as BMI, BP, and lipid levels). Several metabolites with unknown clinical relevance were correlated with signatures consisting of clinical biomarkers indicative of good health, such as 1-(1-enyl-palmitoyl)-2-oleoyl-glycero-3-phosphocholine (GPC) and 1-eicosenoyl-GPC, and that of disease risk, such as glutamate, butyrylcarnitine, lactate, 1-stearoyl-2-dihomo-linolenoyl-GPC, and 1-palmitoleoyl-2-oleoyl-glycerol.

#### Stratification of individuals and characterization

To assess the relationship between the health status of individuals and these 22 key biomarkers, we stratified individuals using hierarchical clustering. This clustering resulted in seven subsets of individuals, each with a unique biomarker signature (threshold of 1.65 correlation distance; Fig. 3b, Additional file 1: Figure S3). The membership of the clusters was further examined by studying its distance relative to other clusters (see the “Methods” section; Additional file 1: Figure S4). We observed that the majority of individuals were closest to their own subset. Cluster analysis solves the practical problem of stratifying individuals to subgroups based on shared signatures of these biomarkers. While the individual profiles with unique signatures in general lie on a continuum and the resulting subsets are not fully isolated from each other, this stratification procedure allows for further investigation such as disease prevalence enrichment in these subsets of individuals.

In order to improve the characterization and our understanding of these subsets, we compared each subset using the full set of 1385 features (Additional file 1: Figure S5). We identified 106 features beyond the 22 used to derive the cardiometabolic subsets that were significantly (*p* < 5.1E−06) enriched in at least one subset compared to the others (Fig. 3b, Additional file 1: Figure S5 and Additional file 2: Table S3). Of the 78 features that were also measured in our validation cohort (TwinsUK baseline), 97.8% of the associations discovered between features and subsets had consistent directions of effect in our validation cohort, and 77.8% were statistically significant (replication *p* < 3.9E−04; Additional file 2: Table S3).

Based on the clinically interpretable biomarkers, such as BMI, liver fat, and insulin resistance, associated with each of the subsets (Additional file 3: Supplemental Notes), we consider subsets 1–4 to be the ones with markers consistent with good health (subset 1 being the “healthiest”) and 5–7 as the subsets with markers consistent with disease risk (subset 7 being the most “at-risk”).

#### Disease prevalence in cohort subsets

**Fig. 4:**
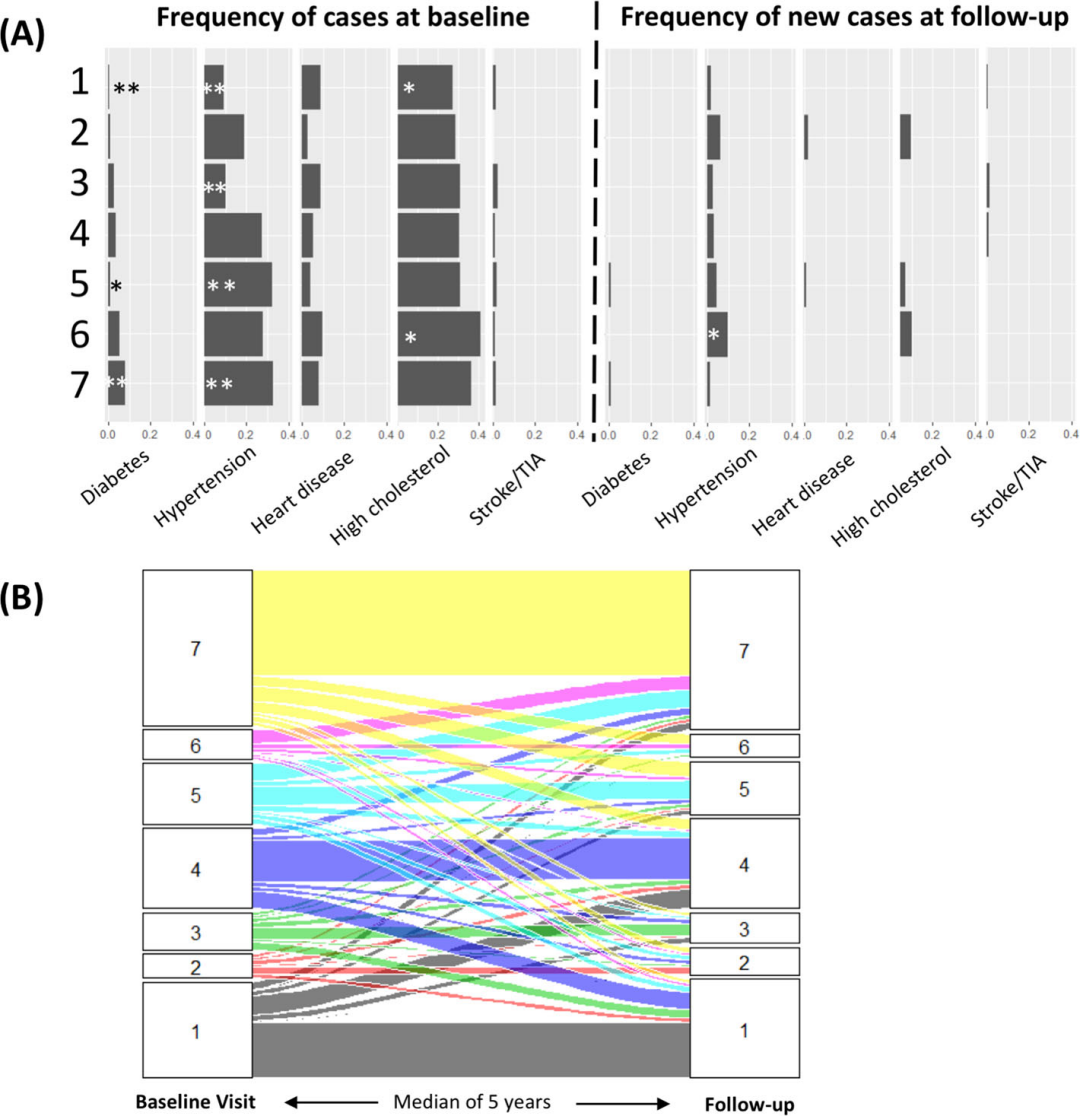
Disease enrichment and longitudinal outcomes of cardiometabolic clusters. **a** Bar plots showing the prevalence of disease at baseline (combined discovery and TwinsUK baseline cohorts; Additional file 1: Figure S2 shows them individually) and the incidence of disease (i.e., only the new cases of disease) after a median of 5.6 years of follow-up (TwinsUK cohort). For Fisher’s exact test comparison of the rate in each cluster vs. the other clusters, *p < 0.05, **p < 0.005. **b** The rates at which individuals from each cluster transition into other clusters after a median of 5.6 years of follow-up. The plot shows individuals per cluster (1 to 7) at baseline visit that transition to other clusters during the follow-up. TIA, transient ischemic attack

In addition to associations with features, we also compared rates of previously diagnosed cardiometabolic conditions between the subsets. We found significant differences between subsets in their rates of diabetes and hypertension diagnoses that were confirmed in the validation cohort (Additional file 3: Supplemental Notes; Fig. 4; Additional file 1: Figure S6). Specifically, subset 7 had significantly higher rates of diabetes, while subset 1 had significantly lower rates of diabetes and hypertension. Interestingly, subset membership was a better predictor of diabetes diagnoses than were the traditional clinical features used to determine diabetes status: glucose, IGT score, IR score, and BMI (Additional file 3: Supplemental Notes). The cardiometabolic key biomarkers that were the largest drivers of this association between diabetes and subset 7 were the IR score, the percent lean body mass, and the metabolites 1-stearoyl-2-dihomo-linolenoyl-GPC (18:0/20:3n3 or 6) and 1-(1-enyl-palmitoyl)-2-oleoyl-GPC (P-16:0/18:1).

We additionally investigated enrichment of rare pathogenic variants in any of the subsets and found only three individuals with such variants (Additional file 3: Supplemental Notes).

#### Longitudinal disease outcome

Our validation cohort was followed for a median of 5.6 (range 1.2–10.1) years, providing us with the opportunity to examine the longitudinal health trends in each subset. During this follow-up, we observed 2 new diagnoses of diabetes, 2 cardiovascular events (angina and myocardial infarction), 7 strokes or transient ischemic attack (TIA), 24 new cases of hypertension, and 37 new cases of hypercholesterolemia. We found a significant difference between subsets in the number of new hypertension cases (Fisher’s exact *p* = 0.009). Specifically, those in subset 6 were at higher risk for developing hypertension, and this association remained significant after controlling for baseline BP, BMI, and age (logistic regression *p* = 0.002).

We also examined subset membership at the follow-up (Fig. 4). We found that subset membership was fairly stable longitudinally, with 51.1% of individuals staying in the same subset at the follow-up visit. For each subset except subset 6, the most common outcome at the follow-up visit was to remain in the same subset. Subset 6 had a very different pattern, with 84.3% of its members transitioning to other subsets, of which 55.8% moved to subset 7. As subset 7 is the one most consistent with poor health in terms of obesity, hypertension, and diabetes, this propensity of subset 6 individuals to transition into subset 7 individuals overtime supports the idea of subset 6 membership as an early precursor to a poor health outcome. Indeed, rates of hypertension were not significantly enriched in subset 6 in the TwinsUK cohort at baseline but were after follow-up. Our analysis therefore supports the classification of subset 6 individuals as being at-risk and prioritized for intervention before they progress to the disease state. However, this classification requires further assessment, especially as our analysis also showed that individuals in subset 6 and 7 should be thought of more as part of a continuous distribution than as two dichotomous groups (Additional file 1: Figure S3).

### Microbiome richness module

The microbiome richness module in the association network contained 167 features, the majority of which were from the metabolome (*n* = 98) and microbiome (*n* = 49) modalities. Similar to the in-depth analysis for the cardiometabolic module, we performed a network analysis to identify key biomarkers of this module and stratified individuals into subsets to assess their health status. Since microbiome was only measured for the last visit in our longitudinal validation cohort, we were unable to perform longitudinal disease outcome analysis for this module.

#### Network analysis for key biomarker selection

**Fig. 5:**
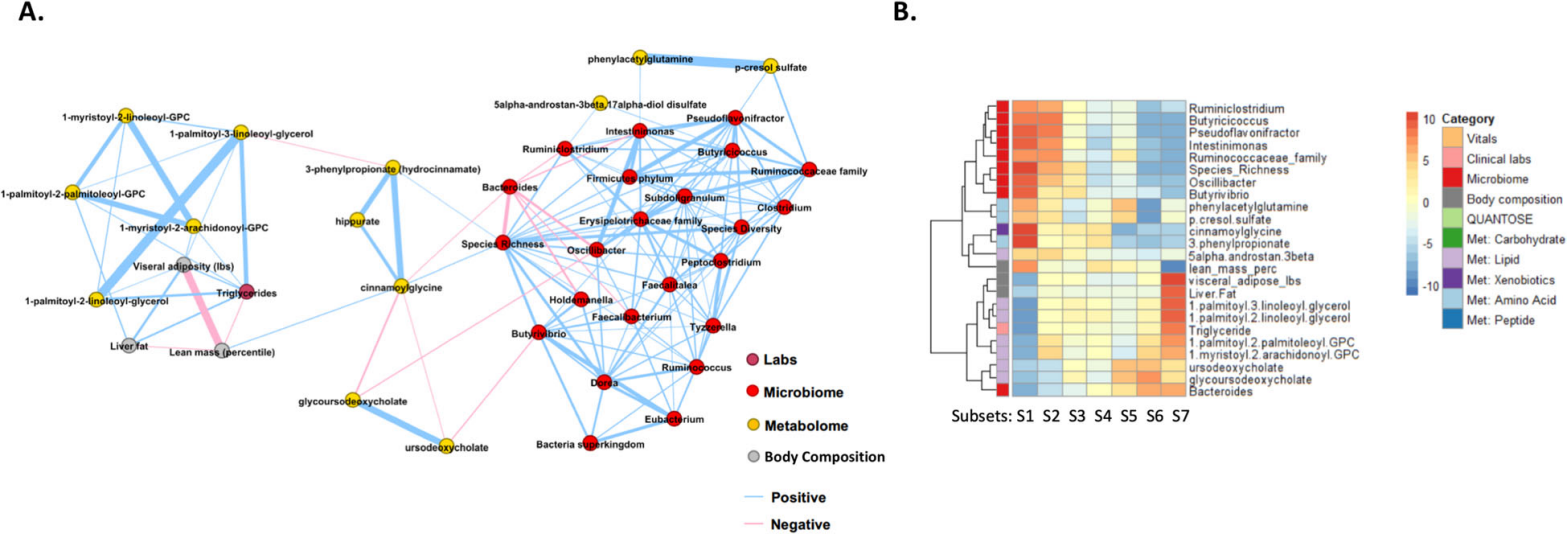
The microbiome richness module. **a** We built a Markov network to identify the key biomarker features that represent the microbiome richness module. Most of the associations between the microbiome and the metabolome were mediated by species richness. **b** We clustered individuals using the key biomarkers. The heatmap shows *z*-statistics from logistic regression for an association between each cluster and each feature. The plot on the left shows the 24 key biomarkers representing the module. Met, metabolome

We construct a Markov network that identified the interface between the microbiome taxa and the metabolites in this module (Fig. 5a). In particular, we observed that most of the associations between the microbiome and the metabolome were mediated by species richness (i.e., the number of species present at a relative abundance greater than 10^−4^). Specifically, species richness is associated to the mutually connected metabolites cinnamoylglycine, hippurate, and 3-phenylpropionate. This relationship is in agreement with a previous study [32] that showed cinnamoylglycine and hippuric acid were not found in germ-free mice, and that 3-phenylpropionic acid is a metabolic product of anaerobic bacteria. Furthermore, a recent study [29] identified hippurate and 3-phenylpropionate as metabolic markers for microbiome diversity, with hippurate being the strongest of the three. These studies indicate that cinnamoylglycine, hippurate, and 3-phenylpropionate are produced by the microbiome. Our model shows a connection between the levels of these metabolites, species richness, and other markers of a healthy metabolome as well as markers of good health, such as low levels of liver fat and visceral adipose tissue. Our findings suggest that species richness is more directly associated with these metabolic markers than species diversity. In addition, cinnamoylglycine may be a metabolic marker for gut microbiome health and the overall health.

As in the case of the cardiometabolic module, we selected our key biomarkers by excluding features that were only connected to their own modality in the Markov network. This resulted in 24 key biomarkers.

#### Stratification of individuals and characterization

Using the 24 key biomarkers, we stratified individuals into 7 subsets (Fig. 5b). The lipid signature that characterized this module had the lowest levels in subset 1 and the highest levels in subset 7, while the microbiome genera abundances and species diversity were the highest in subset 1 and the lowest in 7. The exception was *Bacteroides*, which showed the opposite trend. Associations with the full set of 1345 features showed that subset 7 could be characterized as having markers consistent with being the least healthy, with the highest levels of body fat, BMI, triglycerides, and total cholesterol and the lowest lean mass. Subset 1 had values at the opposite extreme for each of these traits and can be characterized as having markers consistent with the best health. In addition, the subsets were largely distinguished by differences in various lipids and microbiome genera (Additional file 3: Supplemental Notes; Fig. 5b; Additional file 1: Figure S7).

While the subsets could potentially reflect different states of gut microbiome health, which may be associated with overall cardiometabolic health, we found no enrichment of cardiometabolic or other diseases in any of the subsets.

### Comparing membership across the modules

We proceeded to compare the membership of individuals in the subsets from the cardiometabolic and the microbiome richness modules. There was significantly (*p* < 0.001) more overlap of individuals between subsets 7 in the two modules and also between subsets 1 than expected by chance: 66% of those in the microbiome richness subset 7 were in the cardiometabolic subset 7, and 45% of those in the microbiome richness subset 1 were also in the cardiometabolic subset 1. In contrast, only 1% of those in microbiome richness subset 7 were in cardiovascular subset 1 (Additional file 1: Figure S8).

## Discussion

We analyzed 1385 multimodal features collected from 1253 individuals using a combination of unsupervised machine learning and statistical approaches. We identified novel associations and novel biomarker signatures that stratified individuals into distinct health states. The main findings were replicated in an independent validation cohort of 1083 females (TwinsUK). In addition, we showed that such an approach can be used on longitudinal data to identify individuals who may be in the early disease transition state.

Specifically, we performed association analysis of features across modalities and found novel significant associations between *p-*cresol sulfate (pCS) and the microbiome genera *Intestinimonas* and an unclassified genus in the *Erysipelotrichaceae* family. pCS is a known microbial metabolite and is considered to be an uremic toxin [31, 33–35]. It is produced by bacteria fermenting undigested dietary proteins that escape absorption in the small bowel [36–38]. It appears to be elevated in the sera of chronic kidney disease (CKD) patients, and it is associated with increased mortality in patients with CKD [39] and an increased risk of cardiovascular events [39]. The *Intestinimonas* genus is known for being a butyrate-producing species that digests lysine and fructoselysine in the human gut [40], but it is otherwise not well described. Members of the *Erysipelotrichaceae* family might be immunogenic and can potentially flourish after treatment with broad spectrum antibiotics [41]. An increased abundance of *Erysipelotrichaceae* has been observed in obese individuals, and several other lines of evidence suggest a role in lipid metabolism [41]. Our novel associations were validated in the TwinsUK cohort and could further be studied as potential therapeutic targets to decrease pCS levels and its toxicity.

Community detection analysis of the 11,537 statistically significant feature associations identified 2 primary modules of densely connected features: the cardiometabolic module and the microbiome richness module. Both of these modules identified individuals with markers consistent with better health, according to clinical features such as BMI and BP, and individuals with markers consistent with disease risk. Interestingly, when stratifying individuals with distinct signatures in each module together into subsets, the subset of the cardiometabolic module with the markers most consistent with “good” health largely overlapped the microbiome richness subset with the markers that were most consistent with “good” health. The same was observed for the subset with the markers most consistent with disease risk. Such co-enrichment of individuals in the subsets with markers that were most consistent with disease risk derived from both modules suggests patterns of comorbidity and highlights the interaction between cardiometabolic health and gut microbiome health.

The key biomarkers identified in the cardiometabolic module consisted of potentially novel features in addition to the traditional clinical features from several modalities. The potentially novel biomarkers included the abundance of the microbiome genera *Butyrivibrio* and *Pseudoflavonifractor* and several metabolites, such as 1-(1-enyl-palmitoyl)-2-oleoyl-GPC, 1-eicosenoyl-GPC, glutamate, and 1-stearoyl-2-dihomo-linolenoyl-GPC. The higher abundance of the two microbiome genera has been associated with decreased adiposity and improved insulin sensitivity. The *Butyrivibrio* genus is known for its butyrate-producing species and plays a major role in fiber and other complex polysaccharide degradation [42, 43]. An increased abundance of *Butyrivibrio* increases the rate of butyrate production, which is suggested to decrease risk of type 2 diabetes and decreased adiposity [44–46]. In addition, the oral administration of a *Butyrivibrio* species was shown to reduce putative preneoplastic lesions in mice, suggesting a role for the microbiome species as a probiotic in the prevention or suppression of colorectal cancer [44]. A weight-loss study showed enrichment of *Pseudoflavonifractor* at baseline in individuals who succeeded in losing their weight consistently for 2 years [47]. In our study, we observed a higher abundance of *Butyrivibrio* and *Pseudoflavonifractor* in individuals in subset 1, which is consistent with our observation of a very low prevalence of diabetes, hypertension, and obesity in that subset.

We identified another potential biomarker for health from the analysis of the microbiome richness module—the metabolite cinnamoylglycine was associated with microbiome species richness and lean mass percentage. It was observed to be abundant in individuals in subset 1, representing individuals with markers consistent with good health. Cinnamoylglycine is related to gut bacterial metabolism, and it was identified as being present only in the serum or colonic lumen from conventional but not germ-free mice [32]. Additional study is needed to confirm the role of cinnamoylglycine on health and to understand its biological mechanism.

We found that the subset membership for individuals was a better predictor of diabetes than the traditional clinical biomarkers such as glucose, BMI, and insulin resistance. The novel biomarkers in the diabetes signature included 1-stearoyl-2-dihomo-linolenoyl-GPC and 1-(1-enyl-palmitoyl)-2-oleoyl-GPC. These lipid metabolites are not well studied but are likely present in cell membranes and fat-carrying vehicles such as HDL. A study on a related metabolite 1-palmitoyl-2-oleoyl-sn-GPC (POPC) suggested a role in insulin resistance [48]; glucose uptake in skeletal muscle showed that a synthetic reconstituted discoidal HDL made with POPC produced insulin-like effects. Future work on these metabolites may prove them to be novel biomarkers for insulin resistance and diabetes.

A longitudinal disease outcome analysis in the follow-up TwinsUK data found a potential early disease signature for hypertension: membership in the cardiometabolic module subset 6. We also observed that more than half of the individuals from subset 6 transitioned to subset 7, the subset with markers most consistent with disease risk, in the follow-up visit, suggesting that subset 6 membership is an early indication of a poor health outcome. However, we add the caveat that we found subsets 6 and 7 to represent more of a more continuous distribution than two dichotomous groups, which casts some uncertainty onto the utility of separating out subset 6 as their own predisposition group (Additional file 2: Figure S3). Further validation of these signatures is needed to show their utility in prioritizing individuals for intervention.

We did not observe a substantial number of significant findings for the genetic features, which included polygenic risk scores (PRS), HLA types, and known rare disease-causing variants (Additional file 2: Table S3; Additional file 3: Supplementary Notes). This result is not unexpected given the relatively small sample size considered here compared to the large sample sizes required for finding statistically significant association in genetic studies. Additionally, the analyses focus on the main/strongest findings from unsupervised pattern detection, and an overwhelming signal from other functional measurements dampens signals from genetics. The types of associations with the largest effect sizes would be for rare variants and diseases, for which any population-based cohort like the one studied here would be underpowered. Finally, the PRS derived using common variants for certain traits could only explain a small fraction of the variance; therefore, we are underpowered to detect significant associations.

In recent years, several organizations have begun gathering cohorts with high throughput data from multiple modalities. The collection of such datasets from large cohorts is a necessary step in systems medicine to gain comprehensive insights into an individual’s health status and to understand complex disease mechanisms. A systematic and supervised approach to analyze an individual’s genome and deep phenotype data, as shown in our previous publication [4], is important for precision medicine screening. However, it is also crucial to perform unsupervised multimodal data analyses, as described here, to sift through this wealth of information for novel findings of signatures of health and disease. These novel discoveries and the characterization of complex interactions allow us to transition towards personalized, preventative health risk assessments.

## Conclusion

In summary, the approach described in this study demonstrates the power of utilizing a combination of unsupervised machine learning methods on integrated multimodal data to derive novel biomarker signatures for different health states. Additionally, we show application of this approach on longitudinal data to identify potentialearly disease signatures that can stratify individuals for a personalized, preventative health risk assessment.

## Supplementary information

**Additional file 1: Figure S1**. Modularity score. **Figure S2**. Consensus matrix. **Figure S3**. Stratification of individuals. **Figure S4**. Subset membership. **Figure S5**. The cardiometabolic module. **Figure S6**. Prevalence of disease diagnoses. **Figure S7**. The microbiome richness module. **Figure S8**. Membership comparison. **Figure S9**. Network using Graphical Lasso as an alternative method. **Table S1**. Features per modality. **Table S2**. Microbiome genera associated with p-cresol sulfate.

**Additional file 2: Table S3**. Features associated with cardiometabolic subsets.

**Additional file 3.** Supplementary Notes.

**Additional file 4.** Multimodal correlation data.

**Additional file 5.** Median value of key biomarkers, sample subset membership and sample similarity score for the cardiometabolic module and the microbiome richness module.

## Data Availability

The correlation data analyzed in this study is included in this published article as a supplementary file (Additional file 4). The median key biomarker values for each subset, subset membership, and sample similarity score for the cardiometabolic module and the microbiome richness module are provided in Additional file 5. The validation cohort is accessible through managed access in accordance with TwinsUK data governance at http://twinsuk.ac.uk/resources-for-researchers/access-our-data/. We do not have the approval in our IRB protocol to deposit the raw data from our participants, but additional sample-level data are available on reasonable request.

## Abbreviations

BMI: Body mass index
BP: Blood pressure
CAC: Coronary artery calcium
CKD: Chronic kidney disease
CT: Computed tomography
DEXA: Dual-energy x-ray absorptiometry
FDR: False discovery rate
GGT: Gamma-glutamyl transferase
GPC: Glycero-3-phosphocholine
HDL: High-density lipoprotein
HLA: Human leukocyte antigen type
IGT: Impaired glucose tolerance
IR: Insulin resistance
LDL: Low-density lipoprotein
MRI: Magnetic resonance imaging
pCS: *p*-Cresol sulfate
PRS: Polygenic risk scores
STR: Short tandem repeats
TIA: Transient ischemic attack
WGS: Whole genome sequencing
