# Supplementary material for: An unsupervised learning approach to identify novel signatures of health and disease from multimodal data
Source: Genome Med. 2020 Jan 10;12:7. doi: 10.1186/s13073-019-0705-z (PMC6953286; doi:10.1186/s13073-019-0705-z)
Supplement: Supplementary file 1 — Additional file 1: Figure S1. Modularity score. Figure S2. Consensus matrix. Figure S3. Stratification of individuals. Figure S4. Subset membership. Figure S5. The cardiometabolic module. Figure S6. Prevalence of disease diagnoses. Figure S7. The microbiome richness module. Figure S8. Membership comparison. Figure S9. Network using Graphical Lasso as an alternative method. Table S1. Features per modality. Table S2. Microbiome genera associated with p-cresol sulfate. [file 13073_2019_705_MOESM1_ESM.docx]

## Fig S1: Modularity Score

Histogram of modularity score for 300 runs of the (randomized) Louvain community detection algorithm on the cross-modality association network. The average modularity score was 0.369.

## Fig S2: Consensus Matrix

(a) Heatmap for the consensus matrix for the community detection algorithm (run 300 times with different seeds). We notice that the two largest modules, corresponding to the microbiome richness module and the cardiometabolic module, are mostly black, indicating that features in these modules are consistently placed in the same module. (b) By focusing on the submatrix of the consensus matrix corresponding to the 22 key biomarkers extracted from the cardiometabolic module (and later used for stratifying the cohort), we see that these features are consistently placed in the same module. The average the average consistency score (i.e., how often a random pair of them lie in the same module) is 0.85. (c) By focusing on the 40 features from the microbiome richness module with highest centrality score, we see that they are consistently placed in the same module. The average consistency score for these features is 0.93.

## Fig S3: Stratification of Individuals

**
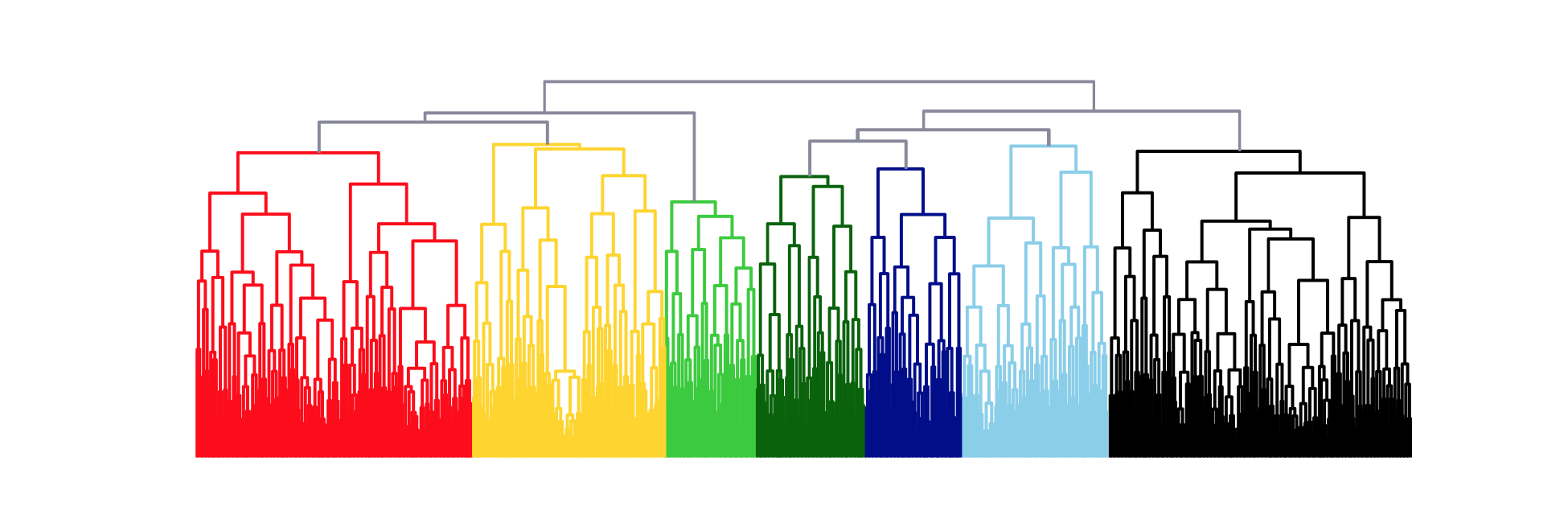
**

Dendrogram obtained from hierarchical clustering of individuals on the 22 key biomarkers selected from the cardiometabolic feature module. We chose the lowest cutoff point (1.68 correlation distance) for which all resulting subsets would have at least fifty individuals. This resulted in the seven clusters (shown in different colors).

## Fig S4: Subset Membership

For each of the seven subsets obtained from the cardiometabolic features, we computed a median profile (i.e., the median score for each of the 22 key biomarkers). For each individual, we found the closest median profile (based on correlation distance). The plot above shows, for each of the seven subsets, the fraction of the individuals with a given closest median profile. We note that, for all seven subsets, most individuals were matched with the median profile of their own subset. We point out that there is a large number of individuals in subset 6 that are closer to the profile of subset 7 than the profile of subset 6 itself.

## Fig S5: The cardiometabolic module


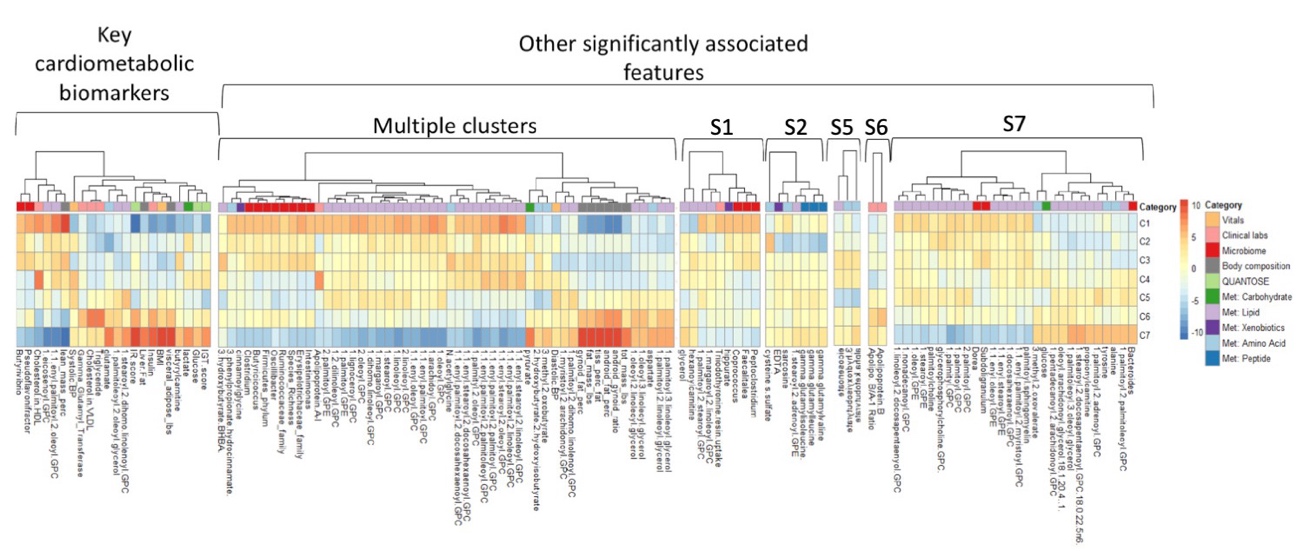


The heatmap shows the Z-statistics from a logistic regression for an association between each subset and each feature. The plot on the left shows the 22 key cardiometabolic features. The plots on the right show significant associations that emerged from an analysis against the full set of 1,385 features. The first plot begins with the features that had significant associations with multiple subsets, and the remaining plots show features that were significantly associated with only one feature. The highlighted groups (e.g., lipid group 1) are largely internally redundant and were collapsed in Figure 4 by plotting their mean Z-statistics value. With the exception of 51 features that were not measured in the TwinsUK cohort (see Table S3), all associations showed directions of effect in the TwinsUK cohort that were consistent with the original association except for the associations between fat mass and visceral adipose and subset 6 (S6), 1-stearoyl-2-docosapentaenoyl-GPC and subset 7(S7), and 3-methyl-2-oxobutyrate and subset 5 (Table S3).

## Fig S6: Prevalence of disease diagnoses


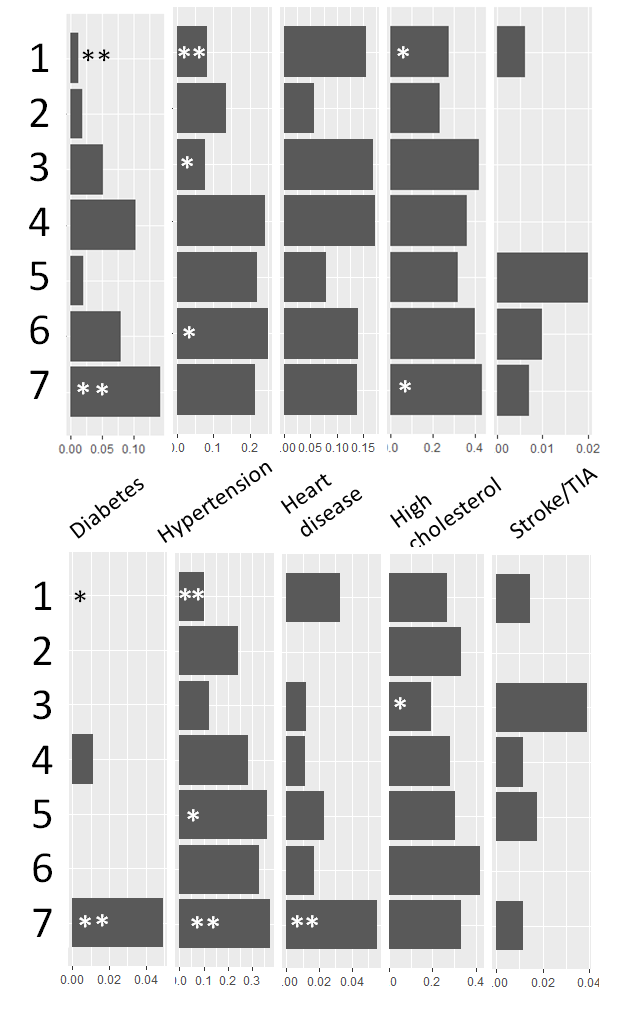


A) Discovery cohort, B) TwinsUK cohort at baseline. The combined cohort is shown in Figure 4A. For Fisher’s exact test comparison of the rate in each subset vs. the other subsets, **p*<0.05, ***p*<0.005. TIA = Transient Ischemic Attack.

## Fig S7: The Microbiome Richness Module


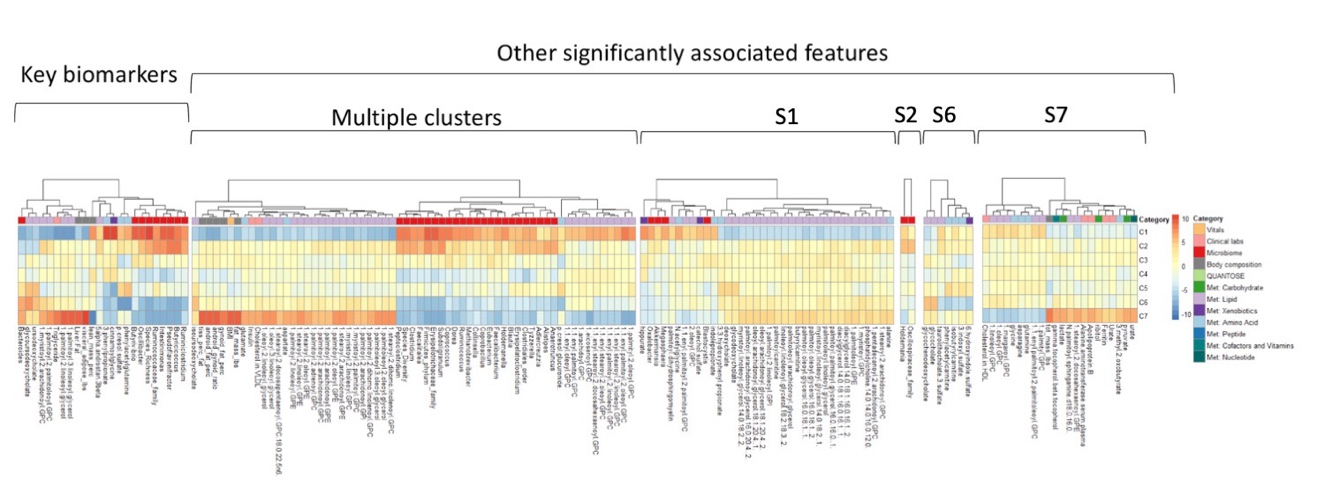


The heatmap shows the Z-statistics from a logistic regression for an association between each subset and each feature. The plot on the left shows the 24 key biomarkers. The plots on the right show significant associations that emerged from an analysis against the full set of 1,385 features. The first plot begins with the features that had significant associations with multiple subsets, and the remaining plots show features that were significantly associated with only one subset.

## Fig S8: Membership Comparison


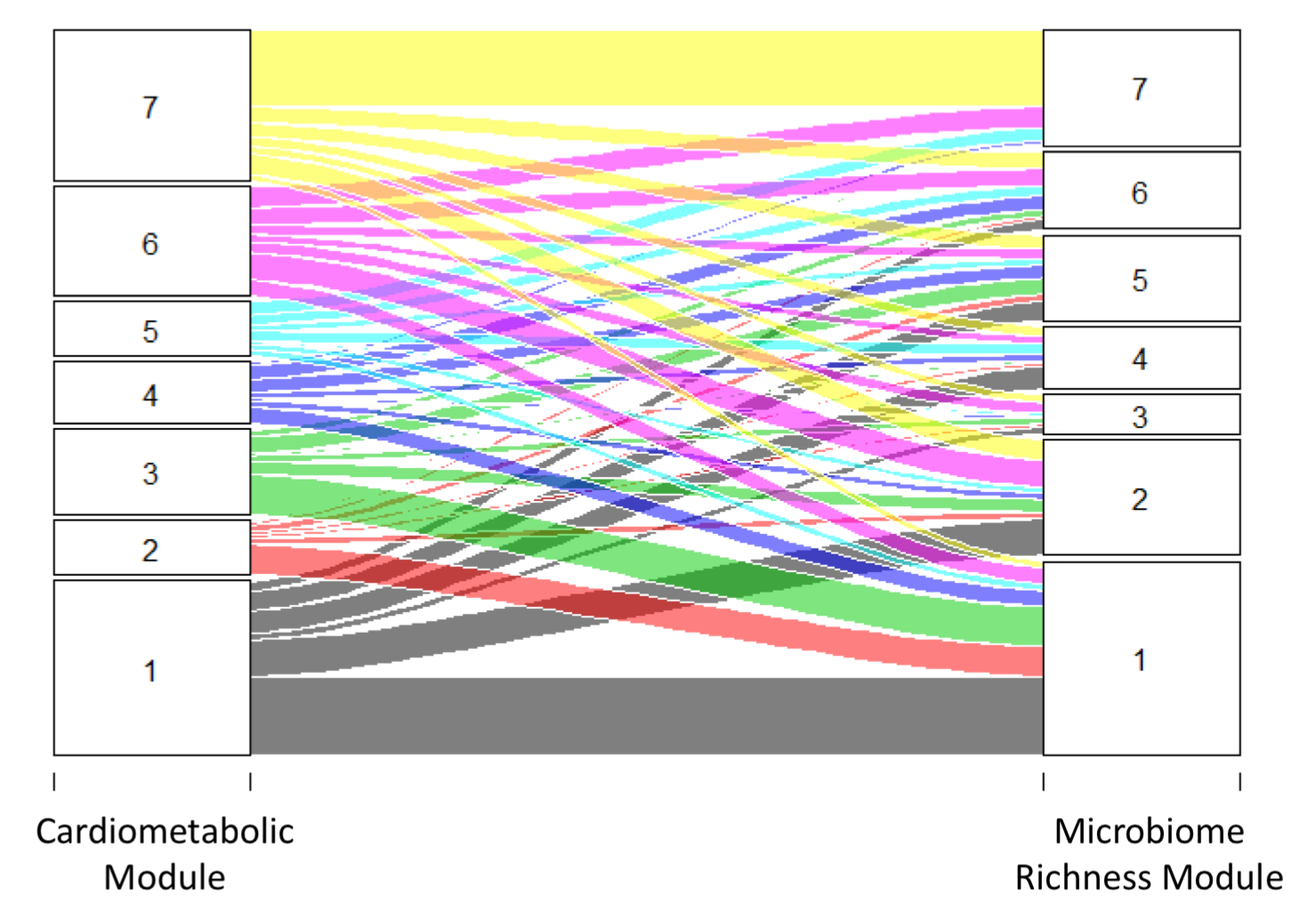


Overlap of subsetted individuals between the cardiometabolic module and the microbiome richness module. There was a high overlap between the two subsets 1 and the two subsets 7. Of those in microbiome richness subset 7, 66% were also in the cardiometabolic subset 7, and 45% of individuals in subset 1 of the microbiome richness module were also members of subset 1 of the cardiometabolic module. In contrast, only 1% of those in microbiome richness subset 7 were in cardiovascular subset 1.

## Fig S9: Network using Graphical Lasso as an alternative method


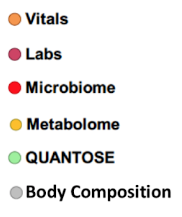


# Network obtained by performing Graphical Lasso on the entire set of features (after performing mean imputation and mapping each feature to a normal distribution). We notice that features from the same modality tend to cluster together. The large number of features makes it difficult to interpret the resulting network, highlighting the value of our two-step approach, where we first perform community detection on the network of cross-modality associations obtaining several feature modules, and then we run Graphical Lasso on the individual modules.

#

## Table S1. Features per modality

| **Modalities** | **Number of individuals** | **Number of features** |
| --- | --- | --- |
| Genetics | 1253 | 182 |
| Personal/family medical history | 1253 | N/A |
| Vitals | 1253 | 4 |
| MRI | 1134 | 5 (Brain) + 4 (Body) |
| Microbiome | 877 | 76 |
| Clinical labs | 769 | 118 |
| Metabolome | 674 | 1010 |
| Quantose | 711 | 2 |
| Body composition | 605 (DEXA) + 1134 (MRI) | 11 |
| CT | 386 | 2 |

The table shows number of individuals and number of features measured per modality. We combined body composition features from DEXA and MRI and treated them as a separate modality (“Body composition”). MRI = magnetic resonance imaging; DEXA = dual-energy X-ray absorptiometry; CT = Computed tomography.

## Table S2. Microbiome genera associated with p-cresol sulfate

| **Microbiome Genus** | **Microbiome Family** | **Association P-value in discovery cohort** | **Association P-value in replication cohort** |
| --- | --- | --- | --- |
| Intestinimonas | Unclassified Clostridiales | 2.92E-24 | 8.54E-12 |
| Unclassified genus in Erysipelotrichaceae family | Erysipelotrichaceae | 2.98E-20 | 3.42E-04 |
| Pseudoflavonifractor | Ruminococcaceae | 9.52E-32 | 1.04E-09 |
| Anaerotruncus | Ruminococcaceae | 1.39E-23 | 1.85E-07 |
| Subdoligranulum | Ruminococcaceae | 9.49E-19 | 2.20E-06 |
| Unclassified genus in Ruminococcaceae family | Ruminococcaceae | 1.95E-38 | 1.20E-14 |
| Ruminiclostridium | Ruminococcaceae | 3.26E-11 | 6.06E-06 |

The table shows microbiome genera that are associated with a metabolite *p*-cresol sulfate in both the discovery cohort and the replication cohort.
