# Supplementary material for: An unsupervised learning approach to identify novel signatures of health and disease from multimodal data
Source: Genome Med. 2020 Jan 10;12:7. doi: 10.1186/s13073-019-0705-z (PMC6953286; doi:10.1186/s13073-019-0705-z)
Supplement: Supplementary file 3 — Additional file 3. Supplementary Notes. [file 13073_2019_705_MOESM3_ESM.docx]

# **Supplementary Notes**

## **Data and Methods Overview**

We collected data from 1,253 self-assessed healthy adults (median age 53; 63% male) across several modalities (Figure 1A), including whole-genome sequencing (WGS)[1], microbiome[2], global metabolome[3], laboratory-developed tests for insulin resistance and prediabetes (Quantose™ [4]), magnetic resonance imaging (MRI), computed tomography (CT) scan, routine lab work, vitals and personal/family medical history. Not all individuals were measured for all modalities (Table S1). For each of the modalities, several data features were measured, totaling 1,385 features from all modalities (see Methods). A majority of the cohort were of European ancestry (71.6%). The remainder were of East Asian (6.4%), Central/South Asian (3.4%), Middle Eastern (0.4%), African (0.3%), and admixed ancestries (18.0%).

We performed four primary analyses using the collected multimodal data as summarized in Figure 1. First, we identified statistically significant associations across the data modalities (Figure 1B). Second, we analyzed the structure of the resulting correlation network by forming “modules” (Figure 1C). Third, we performed an in-depth analysis of selected modules using probabilistic graphical models to identify a “network” of key biomarkers that represents the module (Figure 1D). Fourth, using the key biomarkers, we stratified individuals to partition the study cohort into distinct health profiles with corresponding biomarker signatures (Figure 1E). We further characterized the subsets and examined disease risk using individuals’ personal history and, when available, longitudinal disease diagnosis data. We validated our main findings using an independent TwinsUK validation dataset derived from 1,083 females.

## **Multimodal Correlations**

We calculated correlations for each cross-modality pair of normalized features and selected a list of 11,537 statistically significant associations (with the FDR controlled at 5%) out of 427,415 total cross-modality comparisons. The most significant associations identified, apart from those between metabolome and labs, were expected correlations supporting well-established prior clinical research. Examples include associations between body mass index (BMI) and liver fat percentage (*p* = 1.35E-46) and between visceral adipose tissue (VAT) and insulin resistance (IR) score (*p* = 2.09E-44). These correlations highlight the importance of preventative medicine recommendations for reducing BMI and VAT, which are known risk factors for diabetes and other metabolic syndromes. We also observed height and polygenic risk scores (PRS) for height to be significantly correlated (*p* = 2.32E-44), highlighting the utility of genetics for trait prediction. Other significant genetic associations were observed between the PRS of lipid levels (high density lipoprotein, low density lipoprotein, total cholesterol, and triglyceride) and their corresponding lab measurements. Overall, we observed less than one percent of associations with genetic features that were significant. Conversely, body composition features (e.g. BMI, VAT, android/gynoid ratio, fat mass and lean mass) had the highest percentages of significant associations with several modalities (Figure 2A).

## **Network Robustness Analysis**

For the Louvain community detection method, we accessed the robustness and convergence of the resulting modules. We ran the algorithm 300 times with different seeds, which is used to order the nodes for community expansion. The average modularity score was 0.37 (ranged from 0.36 to 0.38; Figure S1), which was similar to the modularity score achieved by Price et al. [6]. We then built a consensus matrix[5] by calculating the number of times the same pair of nodes are grouped together in a module, across the 300 runs (Figure S1). The features were consistently part of the same module, particularly for the two largest modules (Figure S2).

Additionally, we calculated consistency score (CS) for both sets of key biomarkers identified in the two modules, by counting the average number of times a pair of features were grouped in the same module. The CS was high for both the 22 key biomarkers of the cardiometabolic module and 24 key biomarkers of the microbiome richness module (0.85 and 0.93 , respectively). This finding is also confirmed by the submatrices of the consensus matrix corresponding to the key biomarkers chosen from the cardiometabolic module and from the microbiome diversity module. In the Figure S2, the darker color (higher score) in the matrix implies that the features are consistently placed in the same module by the randomized community detection algorithm.

## **Markov Network Analysis**

While the module identified from the community detection algorithm provides a general overview of how these features are interconnected, its construction is based only on pairwise associations. As such, it contains a significant amount of redundancy (e.g., two metabolites from the same pathway are likely to be connected to the same features from other modalities) and transitive edges. More precisely, if two features A and B are correlated through a third intermediate feature C, the Markov network will not have an edge between A and B. This allows us to identify, for example, which microbiome genera are most strongly connected with metabolome features (and exclude those genera that are connected to metabolome makers *through* other microbiome genera). In order to obtain a more meaningful representation of the interaction between the features in the module, we selected the most central features and computed the inverse covariance matrix. This matrix defines a new network (called the Markov network) on these features with the property that features A and B are only connected if they are correlated conditioned on all other features. This method tends to be less sensitive than the pairwise Spearman associations used to construct the initial network for community detection. Several weaker cross-modality associations were not observed in the Markov network.

## **Cardiometabolic Module**

### **Stratification of Individuals and Characterization**

The individuals in subset 1 can be characterized as containing the individuals with the markers most consistent with good health, with a markedly higher lean mass percentile and low IR score. This subset is also notable for its lower blood pressure, lower butyrylcarnitine levels, and higher HDL. The IR score and lean mass percentile for subsets 2 and 3 indicated individuals with markers consistent with somewhat worse health. In addition, subset 2 displays the lowest glutamate values, while subset 3 is characterized by the lowest blood pressure and the highest levels of 3-hydroxybutyrate. Subset 4 is distinguished by an Impaired Glucose Tolerance (IGT) score that is higher than in the other subsets with healthy individuals and high levels of Apolipoprotein-A (Apo-A) and HDL cholesterol. Subset 5 contains largely overweight individuals who nonetheless have low IR scores and low IGT. Subset 6 contains mostly overweight and obese individuals with high android/gynoid ratios and IR scores; the individuals in this subset were specifically characterized by the highest Apo-B, very low-density lipoprotein cholesterol and triglycerides of any subset. Subset 7 contains the individuals with the most markers consistent with poor health, with a high prevalence of obesity, body fat and insulin resistance.

### **Disease Prevalence in Subsets**

In addition to associations with features, we also compared rates of previously diagnosed cardio-metabolic conditions (i.e., diabetes, hypertension, hypercholesterolemia, heart disease, and stroke) between the subsets. We found significant differences between subsets in their rates of diabetes and hypertension diagnoses (Fisher’s exact *p* = 1.0E-04 and 2.3E-04, respectively). The findings were confirmed in the validation cohort (Fisher’s exact *p* = 4.3E-04 and < 1.0E-06, respectively) (Figure 4). Specifically, subset 7 had significantly higher rates of diabetes, while subset 1 had significantly lower rates of diabetes and hypertension. There were no significant differences between the subsets in heart disease or stroke history, though hypercholesterolemia showed a trend toward group differences in both cohorts that requires further validation (*p* = 0.03 in the discovery cohort; *p* = 0.09 in the validation cohort).

Interestingly, subset membership was a better predictor of diabetes diagnoses than were the traditional clinical features used to determine diabetes status: glucose, IGT score, and IR score, as well as BMI. Even after accounting for these traditionally predictive features, individuals in subset 7 were significantly more likely to have diabetes than were members of the other subsets (logistic regression *p* = 0.01). The enrichment of hypertension diagnosis in subset 7, however, was explained by blood pressure measurement, as expected.

The cardiometabolic key biomarkers that were the largest drivers of this association between diabetes and subset 7 were the IR score, percent lean body mass, and the metabolites 1-stearoyl-2-dihomo-linolenoyl-GPC (18:0/20:3n3 or 6) and 1-(1-enyl-palmitoyl)-2-oleoyl-GPC (P-16:0/18:1). In a multivariable logistic regression containing the 22 cardiometabolic key biomarkers, the above mentioned were the four features that were significantly associated with diabetes status. They were also significant predictors of subset 7, in addition to liver fat, HDL cholesterol, *Pseudoflavonifractor*, and the metabolites lactate and 1-eicosenoyl-GPC (20:1)).

We next sought to identify features that distinguished those in subset 7 who did and did not have diabetes. We compared these two groups within subset 7 for all 1,385 features, and the metabolite citrulline emerged as by far the best predictor of diabetes status, with decreased levels of citrulline being found in diabetes patients (*p* = 5.5E-06). We specifically found that this signal was due to diabetic patients on metformin medication, as opposed to other medications, a result consistent with studies showing decrease in serum concentration of citrulline after metformin therapy[7, 8].

We additionally investigated whether individuals with known rare pathogenic variants associated with obesity, cardiovascular conditions, or diabetes were enriched in any of the subsets (see Methods). However, we only found 3 individuals with known pathogenic/likely pathogenic variants: a pathogenic *TTR* variant (rs76992529; p.Val142Ile) for cardiomyopathy in a subset 7 individual, a pathogenic *WFS1* variant (rs71530923; p.Arg42Ter) for Diabetes with insipidus, optic atrophy, and deafness in a subset 1 individual, and a likely pathogenic *HNF1A* variant (rs193922587; p.Leu555Phe) for maturity onset diabetes of the young in a subset 1 individual.

## **Microbiome Richness Module**

### **Stratification of Individuals and Characterization**

The subsets were largely distinguished by differences in various lipids and microbiome genera. The enrichments most specific to each subset were as follows: Subset 1 had the highest levels of hippurate and cinnamoylglycine; Subset 2 had the highest levels of *Holdemania*, *Oscillospiraceae*, *Ruminococcaceae*, and *Alistipes*; Subset 3 had the lowest levels of *Flavonifractor* and 3-methylglutaconate; Subset 4 had the lowest levels of *Lachnospiraceae*; Subset 5 had the highest levels of 4-hydroxyhippurate and 3'-3-hydroxyphenyl propionate and the lowest levels of cinnamoylglycine; Subset 6 had the highest levels of glycoursodeoxycholate and lowest levels of p-cresol sulfate, p-cresol glucuronide, phenylacetylglutamine, 6-hydroxyindole sulfate and *Ruminiclostridium*; and Subset 7 had the lowest levels of HDL and the highest urate, ferritin, Apo-B, and diastolic blood pressure levels.

## **References**

1. Telenti A, Pierce LCT, Biggs WH, di Iulio J, Wong EHM, Fabani MM, et al. Deep sequencing of 10,000 human genomes. Proc Natl Acad Sci U S A. 2016;113:11901–6. doi:10.1073/pnas.1613365113.

2. Anderson EL, Li W, Klitgord N, Highlander SK, Dayrit M, Seguritan V, et al. A robust ambient temperature collection and stabilization strategy: Enabling worldwide functional studies of the human microbiome. Sci Rep. 2016;6:31731. doi:10.1038/srep31731.

3. Guo L, Milburn M V., Ryals JA, Lonergan SC, Mitchell MW, Wulff JE, et al. Plasma metabolomic profiles enhance precision medicine for volunteers of normal health. Proc Natl Acad Sci. 2015;112:E4901–10. doi:10.1073/pnas.1508425112.

4. Cobb J, Gall W, Adam K-P, Nakhle P, Button E, Hathorn J, et al. A novel fasting blood test for insulin resistance and prediabetes. J Diabetes Sci Technol. 2013;7:100–10. doi:10.1177/193229681300700112.

5. Monti S, Tamayo P, Mesirov J, Golub T. Consensus Clustering: A Resampling-Based Method for Class Discovery and Visualization of Gene Expression Microarray Data. Mach Learn. 2003;52 1/2:91–118. doi:10.1023/A:1023949509487.

6. Price ND, Magis AT, Earls JC, Glusman G, Levy R, Lausted C, et al. A wellness study of 108 individuals using personal, dense, dynamic data clouds. Nat Biotechnol. 2017;35:747–56. doi:10.1038/nbt.3870.

7. Breier M, Wahl S, Prehn C, Ferrari U, Sacco V, Weise M, et al. Immediate reduction of serum citrulline but no change of steroid profile after initiation of metformin in individuals with type 2 diabetes. J Steroid Biochem Mol Biol. 2017;174:114–9. doi:10.1016/J.JSBMB.2017.08.004.

8. Adam J, Brandmaier S, Leonhardt J, Scheerer MF, Mohney RP, Xu T, et al. Metformin Effect on Nontargeted Metabolite Profiles in Patients With Type 2 Diabetes and in Multiple Murine Tissues. Diabetes. 2016;65:3776–85. doi:10.2337/db16-0512.
